# Artificial Sweet Cherry miRNA 396 Promotes Early Flowering in Vernalization-Dependent Arabidopsis Edi-0 Ecotype

**DOI:** 10.3390/plants14060899

**Published:** 2025-03-13

**Authors:** José Gaete-Loyola, Felipe Olivares, Gabriela M. Saavedra, Tiare Zúñiga, Roxana Mora, Ignacio Ríos, Gonzalo Valdovinos, Marion Barrera, Andrea Miyasaka Almeida, Humberto Prieto

**Affiliations:** 1 Centro de Genómica y Bioinformática, Facultad de Ciencias, Ingeniería y Tecnología, Universidad Mayor, Santiago 8580745, Chile; jose.gaetelo@umayor.cl (J.G.-L.); gabriela.saavedrao@mayor.cl (G.M.S.); 2 Biotechnology Laboratory, La Platina Research Station, National Institute of Agriculture (INIA), La Pintana, Santiago 8831314, Chile; felipe.olivares@inia.cl (F.O.);; 3 Programa de Doctorado en Genómica Integrativa, Vicerrectoría de Investigación, Universidad Mayor, Huechuraba, Santiago 8580745, Chile; 4 Natural Sciences, Mathematics, and Environment Faculty, Metropolitan Technological University (UTEM), Macul, Santiago 8330526, Chile; tzuniga@utem.cl; 5 Escuela de Agronomía, Facultad de Ciencias, Ingeniería y Tecnología, Universidad Mayor, Huechuraba, Santiago 8580745, Chile

**Keywords:** miRNA, chilling requirement, *Prunus*, dormancy

## Abstract

The flowering and fruiting of sweet cherry (*Prunus avium* L.) depend on precise synchronization with seasonal events. During harsh autumn and winter conditions, floral buds enter dormancy to protect and prepare for the productive season. Dormancy release occurs after exposure to genotype-specific chilling temperatures, an event in which epigenetic reprogramming triggers further metabolic and gene expression activation. Similarly, several *Arabidopsis* ecotypes require chilling (vernalization) to transition from vegetative to floral states. At vernalization’s end, the decrease in the repressor complex formed by *SHORT VEGETATIVE PHASE* (*SVP*) and *FLOWERING LOCUS C* (*FLC*) allows *FLOWERING LOCUS T* (*FT*) to induce flowering. However, this alone does not fully explain the process. MicroRNAs (miRNAs) play a crucial role in gene regulation during plant development and environmental interactions, and miR396’s role during flower development and vernalization has been described in some plant species, although not for sweet cherry dormancy. We used ‘Regina’, a high-chill sweet cherry variety, to identify candidate small RNA molecules throughout dormancy, resulting in the detection of miR396. The transcript expression levels of the putative miRNA target genes were evaluated through quantitative PCR analyses of dormant buds. Additionally, an artificial sweet cherry miR396 was used to transform *Arabidopsis* Edi-0, a vernalization-requiring ecotype. Ectopic expression of this artificial molecule partially mirrored the effect on target genes observed in dormant buds and, more importantly, led to vernalization-independent flowering. Artificial miR396 expression also resulted in decreased *FLC* and increased *SVP* and *FT* transcript levels. These results could pave the way for future studies on the involvement of miR396 in the regulation of dormancy and flowering, with potential applications in improving crop resilience and productivity.

## 1. Introduction

Sweet cherry (*Prunus avium* L.) adult trees bloom in the spring after a period of embryogenic development arrest during winter called dormancy. The transition between dormancy and flowering is influenced by environmental factors and depends on an adequate seasonal timing at which these stages take place [1,2]. The dormancy period allows perennial trees to flower and set fruit under favorable conditions. Floral and vegetative sweet cherry buds develop immediately after fruit harvest and remain in a state known as paradormancy [3]. At this stage, growth inhibition is induced by signal redistribution between organs, primarily related to the phenomenon of apical dominance and hormone redistribution [3]. Shortening of photoperiod and cold temperatures in autumn induce endogenous signals, which lead to a deep stage of dormancy denominated endodormancy [2]. Release from endodormancy requires exposition to a period of chill (measured in hours at 0–7 °C), a process referred to as chill requirement, which is a genotype-dependent trait [4,5]. Once the chilling requirement is fulfilled, buds are allowed to transit from endodormancy to ecodormancy; at this rate, buds remain dormant while cold temperatures persist. When favorable environmental conditions occur, such as warmer temperatures, growth is resumed, leading to flowering [6].

The dormancy process is accompanied by a series of molecular events that include changes in metabolic activities in phenylpropanoid [7] and carbohydrate [8] pathways, hormone fluctuations [9], and changes in the oxidative landscape [10,11]. Comprehensive analyses of genes involved in the dormancy process have recently been carried out [12,13,14], including meta-analyses [15]. An active reprogramming of gene expression appears as a key component of the process. Particularly, members of the gene groups, such as MADS-box transcription factors, hormones (abscisic acid (ABA), gibberellic acid (GA), and auxins) metabolism, gametogenesis, epigenetic regulators, and temperature and light sensing systems, have been associated with high- or low-CR varieties in these species, including *P. avium* [15]. These data are partially consistent with the idea by Zhang et al. [16] that endodormancy is caused by cold induction of *C-REPEAT BINDING FACTOR* (*CBF*) transcripts, which leads to the expression of *DORMANCY-ASSOCIATED MADS-box* (*DAM*) genes and inhibition of the GA signaling pathway, a process that is later reversed by the long-term cold accumulation, reducing *CBF* and *DAM* gene expression.

In contrast, annual plants such as *Arabidopsis thaliana* have meristem determination and floral transition occur in a synchronized and irreversible manner in response to internal and external cues. This flowering plant completes its life cycle in a single growing season and typically has a monocarpic growth, reproduces only once, and dies [17]. In *A. thaliana*, the flowering process is governed by four mechanisms [18,19]: (1) photoperiod, which triggers flowering during extended daylight; (2) vernalization, which prepares the plant for flowering after prolonged exposure to cold (winter); (3) gibberellins, which are necessary to stimulate flowering under non-inductive photoperiods; and (4) the autonomous pathway, a mechanism that operates independently of environmental cues. These pathways collectively modulate the expression of the integrator genes for flowering time, such as *SUPPRESSOR OF OVEREXPRESSION OF CONSTANS1* (*SOC1*) and *FLOWERING LOCUS T* (*FT*), which subsequently activate genes responsible for floral meristem identity, including *LEAFY* (*LFY*) and *ACTIVATOR PROTEIN 1* (*AP1*) [18]. The integrator *FT* encodes for a small protein generated in the leaf vasculature, which travels to the apical meristem to promote expression of floral meristem identity genes. On the other hand, two members of the MADS-box transcription factors family, the *FLOWERING LOCUS C* (*FLC*) and *SHORT VEGETATIVE PHASE* (*SVP*), negatively regulate *SOC1* and *FT* [20]. *FLOWERING LOCUS C* is a potent inhibitor of FT, and during vernalization its promoter is remodeled and silenced by *VERNALIZATION INSENSITIVE 3* (*VIN3*), allowing FT expression. With temperature and photoperiod as main factors in the process, most of the Arabidopsis ecotypes require a prolonged chilling exposition before spring flowering [19,21,22]. Rapid-cycling ecotypes such as Columbia, can flower with no chilling requirement. On the other hand, late-flowering ecotypes, such as Edi-0, are winter-annuals that require vernalization before flowering [23]. These differential vernalization requirements are mainly due to the role of *FLC* and *SVP*. In addition, genetic studies have included *FRIGIDA* (*FRI*) in these differential vernalization requirements among ecotypes by up-regulating *FLC* [19]. Allelic variations at the *FLC* locus determine the Arabidopsis natural variation in vernalization [24]. The early flowering phenotype of spring ecotypes is associated with loss-of-function mutations at the *FRI* and *FLC* loci [25].

The vast array of processes involving metabolic, epigenetic, and gene expression alterations during dormancy suggests the participation of additional factors that link environmental and genetic influences [26,27]. Small RNAs, ranging from 20 to 24 nucleotides (nt), represent key regulators of gene expression affecting all levels of genetic information in plants. Generally divided into two main categories, microRNAs (miRNAs) and small interfering RNAs (siRNAs), these molecules provide sequence specificity through base complementarity for specific target gene recognition by the RNA silencing machinery [28]. MiRNAs, encoded by genes (and transcribed to pri-miRNAs), can regulate both the chromatin state of their targets and the availability of the encoded transcripts for translation into functional proteins [29]. Almost all intracellular processes associated with plant growth and development involve participation of miRNAs [30], and they constitute important regulators of floral and flowering-related phases through post-transcriptional regulation of corresponding genes and transcriptional factors [31,32]. Small RNAs participate in the post-transcriptional regulation of Dormancy-Associated MADS-Box (DAM) genes in fruit trees [26,33,34]. In pear, comprehensive analyses of miRNAs during endodormancy maintenance and release at a genome-wide level revealed transcriptional patterns of these molecules in the process, highlighting, for instance, the degradation of DAM transcripts [34].

Different studies have involved miR396 in flowering processes of annual species (*A. thaliana*) by regulating flower development by targeting growth-regulating factor (GRF) genes [35]. Interestingly, overexpression of miR396 in perennial grasses (*Agrostis stolonifera*) bypasses vernalization requirements for flowering [36]. Micro RNA396 belongs to a group of conserved miRNAs that regulate plant development and hormone signaling [37] by regulating transcription factors that control cell proliferation and organ growth in plants [35]. Different GRF genes have been described to be targeted by this miRNA depending on the plant species and development stage [38]. MiR396 also interacts with other miRNAs and phytohormones to modulate the GRF-mediated responses to environmental stress and developmental signals [39], suppressing the expression either of GRF genes or of GRF-interacting factors [35].

The similarities between dormancy and vernalization present a compelling potential to utilize the annual species as a model for investigating the impact of essential miRNAs in dormancy in perennial trees. We preliminarily employed next-generation small RNA sequencing and verified the presence of miR396 and other miRNAs throughout dormancy in floral buds of ‘Regina’, a high-chill sweet cherry variety. To examine the effects of its ectopic expression during vernalization, we developed an artificial variant of miR396 (amiR396) and utilized this construct for ectopic expression in the vernalization-dependent Arabidopsis Edi-0 ecotype. Molecular findings facilitated comparisons of the status of miR396-target genes in dormant sweet cherry floral buds and in transgenic Arabidopsis individuals. The phenotype produced by amiR396 showed the influence of this molecule on blooming with a vernalization-regulated context, leading to early-flowering occurrence.

## 2. Results

### 2.1. Determination of Dormancy Stages in ‘Regina’

**Figure 1:**
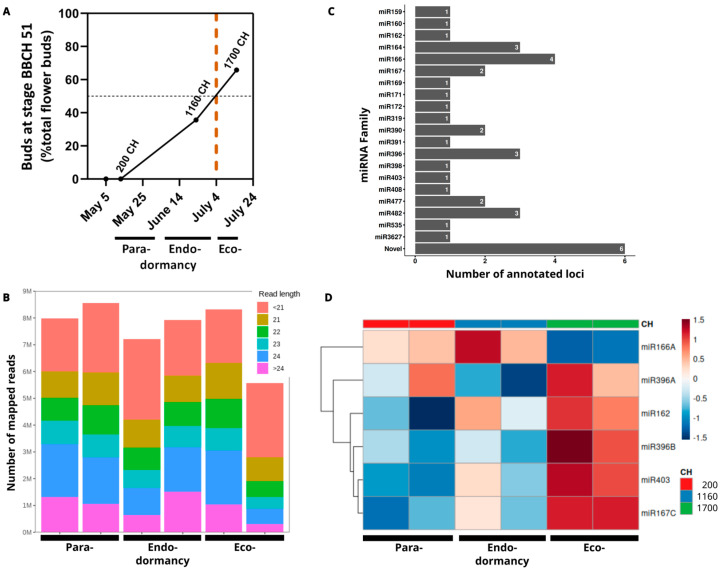
Dormancy status under controlled chilling accumulation and small RNA species produced in ‘Regina’. (**A**) Evaluation of bud burst under forcing conditions for the cultivar ‘Regina’. The dashed and dotted lines represent the dormancy release date, estimated at 50% of buds at BBCH stage 51 [1]. (**B**) Identification of small RNA populations during the chilling accumulation process in floral buds, showing the length of reads mapped to the reference genome in each sample. (**C**) Annotation of miRNA loci identified from small RNA-seq data. (**D**) Heatmap of the differentially expressed miRNAs, expression values were converted to z-scores, where red and blue colors represent up- and down-regulated miRNAs, respectively. Relevant microRNA molecules, target genes, and involved processes.

Bud break percentages under continuous chilling assays over the flower buds were established based on continuous morphological observations using the BBCH scale. The results were as in previous experiments describing cold requirements for this cultivar and ranged from 1000 to 1500 chilling hours (CH) to flowering (Figure 1A) [40]. Three main dormancy stages were defined according to Lang et al. [3], including (1) paradormancy at an early stage of the experiment (200 CH), (2) the endodormancy phase unresponsive to forcing bud-activation conditions (1160 CH), and (3) ecodormancy, when over 50% of the buds exhibit dormancy release under forcing conditions (1700 CH).

The quality assessment process for the small RNA-seq libraries allowed the selection of approximately 70% of the generated data (Table 1). From these reads, 88% were mapped to the reference genome; the candidate small RNA-associated universe was predominantly comprised of 24-nt, followed by 21-nt size molecules (Figure 1B). This alignment led to the identification of 38 miRNA loci; data integration from the reference genome and available miRNA databases enabled the annotation of 20 miRNAs (Figure 1C), while 6 miRNAs showed no matches in the databases and were therefore classified as novel. Among the annotated miRNA families are miR159, miR160, miR162, miR164, miR166, miR167, miR169, miR171, miR172, miR319, miR390, miR391, miR396, miR398, miR403, miR408, miR477, miR482, miR535, and miR3627.

**Figure 2:**
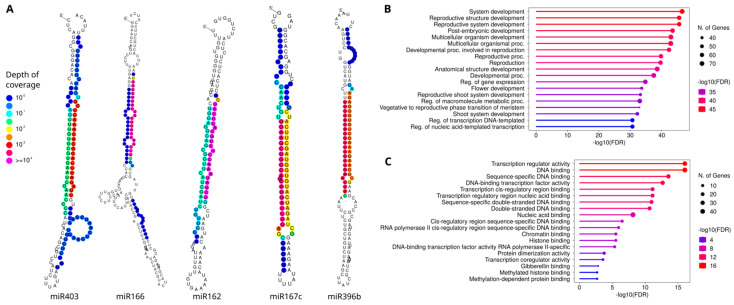
MicroRNAs associated with dormancy and Gene Ontologies (GOs) of their target genes. (**A**) Secondary structure of the associated pre-miRNAs, with colors representing the depth of coverage or the number of reads supporting each specific base. (**B**) GO term enrichment of the target genes for biological processes. (**C**) GO term enrichment of the target genes for molecular functions.

A preliminary identification of the most relevant molecules among this group was carried out by a differential expression analysis from sequencing data obtained under continuous CH conditions. Five miRNAs exhibited statistically significant changes in their expression profiles under the different dormancy stages, as illustrated in Figure 1D. The differentially expressed miRNAs corresponded to miR162, miR166, miR167c, miR396b, and miR403. Except for miR166, which exhibited its highest expression during paradormancy, the other miRNAs displayed an expression pattern characterized by low expression levels at 200 CH and 1160 CH, followed by high expression levels at 1700 CH. The secondary structures of these pre-miRNAs derived from the genomic information available for sweet cherry are shown in Figure 2A, and their functions in other species are detailed in Table 2.

The use of psRNAtarget enabled the prediction of 48 target genes susceptible to inhibition through degradation mediated by these preliminary differentially expressed miRNA molecules. Among them, 16 were transcription factors, including 11 *GROWTH-REGULATING FACTORS* (*GRF*), 4 *HOMOEBOX-LEUCINE ZIPPER* (*HD-ZIP*), and 1 *DNA-BINDING with ONE FINGER* (*DOF*). Gene Ontology (GO) analysis of these target genes revealed significant enrichment in various biological processes (Figure 2B), particularly in the development of reproductive and shoot systems, flower and reproductive structure formation, regulation of gene expression, transcription, and macromolecule metabolic processes. In terms of molecular function, the most enriched categories were associated with binding activities related to regulation of transcription, as illustrated in Figure 2C. These binding functions covered a diverse range, including interactions with transcription factors, cis-regulatory regions, and others related to methylation and histone modifications, indicative of the multifaceted regulatory roles mediated by the identified miRNAs.

Integration of this genomic information into biological functions and pathways by KEGG analysis revealed enrichment of five genes in the circadian rhythm-plant pathway (*HEADING DATE 3A* (*FT*; AT1G65480), *ADAGIO PROTEIN 3* (*ADO3*; AT1G68050), *PROTEIN SUPPRESSOR OF PHYA-105 1-like isoform X1* (SPA1; AT2G46340), *ADO1* (*FT1*; AT5G57360), and *ARABIDOPSIS PSEUDO-RESPONSE REGULATOR* (*TOC1*; AT5G61380) and four genes in the starch and sucrose metabolism (*ALPHA,ALPHA-TREHALOSE-PHOSPHATE SYNTHASE* (*ATTPS1*; AT1G78580), SUCROSE SYNTHASE (*ATSUS4*; AT3G43190), *GLUCOSE-6-PHOSPHATE ISOMERASE 1*, chloroplastic (*PGI*; AT4G24620) and GLUCOSE-A-PHOSPHATE ADENYLTRANSFERASE SMALL SUBUNIT, chloroplastic/amyloplastic (*ADG1*; AT5G48300); all these genes are regulated by miR396. Supplementary Table S1 presents the putative target genes, their proposed functions, and their potential impact on the flowering process of the species. These analyses suggest a significant regulatory network in which the conserved miR396 molecule is especially attractive, considering the inclusion of target genes involving transcription factors, epigenetic-linked processes, and *GROWTH-REGULATING FACTORS* (*GRF*s).

### 2.2. Insights on the miR396 Activity

**Figure 3:**
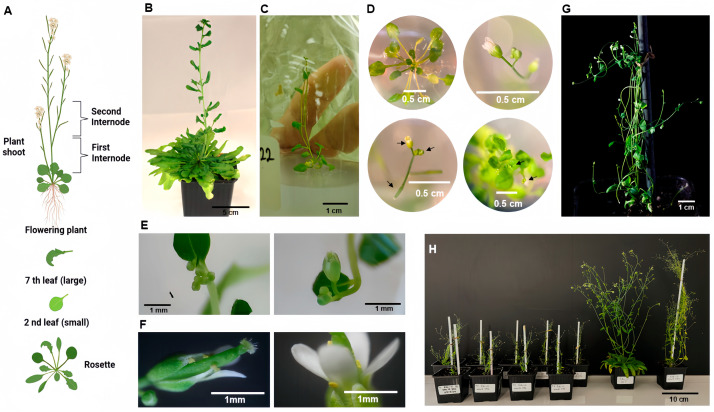
Transgenic Edi-0 *Arabidopsis* lines overexpressing an artificial version of miR396. MicroRNA 396 was investigated for its ectopic expression in the *Arabidopsis* vernalization-requiring Edi-0 ecotype. Individuals expressing the mature artificial version of miR396 (T3) grew until senescence. The principal structures visualized in a wild-type *Arabidopsis* individual are schematized (**A**) and shown for an Edi-0 individual (**B**). Artificial miR396-overexpressing individuals began flowering on average 62 days after sowing (DAS) (**C**), whereas wild-type required 209 DAS. The rosette-like structure in transgenic individuals consisted of only 4–8 smaller, rounded leaves (**C** and **D**-upper-left); at this stage, flowers and floral buds were already formed in these individuals (**D**). The generated flowers did not show abnormalities in shape and/or structure (**D**–**F**). Pods were formed at a very early stage (**D**-lower-left), yielding viable seeds. Artificial miR396-overexpressing individuals exhibited reduced height (**G**,**H**) compared to wild-type individuals and to an additional control in which artificial miR162 was overexpressed in the same ecotype. Details for flowering and rosette structures are indicated in Table 3.

The effect deduced for miR396 led to further examination of its ectopic expression in a vernalization-requiring *Arabidopsis* ecotype. *Arabidopsis* Edi-0 individuals were transformed using an artificial miR396 (amiRNA) construct to specifically produce this mature miRNA molecule. To our surprise, the overexpression of amiR396 resulted in early flowering, occurring as early as 64 days post-sowing, compared to the 229 days required by the wild-type individuals (Figure 3 and Table 3). Some typical structures in an *Arabidopsis* plant are shown in Figure 3A, where rosettes are typically broad and flat, formed by leaves that grow in a circular pattern close to the ground, maximizing light absorption for photosynthesis. Before bolting, leaves are typically oval to lance-shaped with slightly serrated edges (a complete developmental register is shown in Supplementary Figure S1). Wild-type Edi-0 individuals required around 128 leaves in their rosette to initiate flowering (Figure 3B, Table 3). In contrast, miR396-expressing plants formed a rosette-like structure with only 4–8 smaller rounded leaves (Figure 3C and Figure 3D-upper-left; Table 3). We did not observe abnormalities in the shape and structure of flowers (Figure 3D–F), and the generated pods (Figure 3D-lower-left) yielded viable seeds. A complete graphical description for these individuals and other independent lines are shown in Supplementary Figures S2 and S3. Plants expressing amiR396 exhibited reduced height and multiple flowering points (Figure 3G and Figure 3H-left). An additional comparative experiment, in which the artificial miR162 molecules were overexpressed in Edi-0 individuals, showed that this construct did not induce flowering until 146 days after sowing, closely matching the timeline observed in the untransformed Edi-0 plants (Table 3; Figure 3H-right; Supplementary Figures S4 and S5).

**Figure 4:**
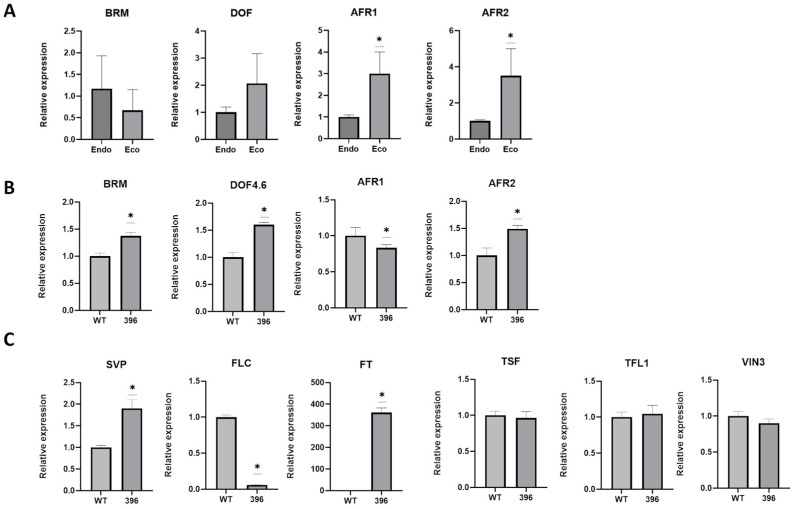
Analysis of relevant genes associated with miR396. (**A**) Expression analysis of potential genes involved in regulatory networks or epigenetic events associated with miR396 in RNA samples from endo- and eco-dormancy sweet cherry floral buds. (**B**) Expression analysis of the same potential genes evaluated in RNA samples obtained from *Arabidopsis* Edi-0 individuals overexpressing the artificial miR396. (**C**) Analyses of transcript levels of genes associated with the key components of the flowering repressor complex in transgenic *Arabidopsis* Edi-0 miR396 lines. For (**A**,**B**), qPCR detections corresponded to *SAP30 FUNCTION RELATED 1* (*AFR1*), *SAP30 FUNCTION-RELATED 2* (*AFR2*), *DNA One Zinc Finger Protein* (*DOF*), and ATP Dependent Helicase *BRAHMA* (*BRM*). For (**C**), qPCR detections corresponded to *SHORT VEGETATIVE PHASE* (*SVP*), *FLOWERING LOCUS C* (*FLC*), *FLOWERING LOCUS T* (*FT*), *TWIN SISTER OF FT* (*TSF*), *TERMINAL FLOWER 1* (*TFL1*), and *VERNALIZATION INSENSITIVE 3* (*VIN3*); TAIR IDs for these selected genes are indicated in Supplementary Table S1. The data were analyzed for raw statistics with ANOVA (two-way) and tested for significant differences (Tukey’s test) using the statistical software package Prisma 10.0.2(232) (GraphPad Software Inc., Boston, MA, USA). Significant differences in expression were calculated using a Mann–Whitney test. The significance (95% probability level) of the difference between means is plotted and represented by an asterisk. The values were averaged over three replicates for each time or condition. Processed data and statistics for each analysis are provided in Supplementary Table S2.

To understand the molecular events underlying the early flowering phenotype of Edi-0 plants overexpressing amiR396, we evaluated the expression status of selected genes predicted as miR396 targets, mostly associated with epigenetic events, in both sweet cherry dormant buds and *Arabidopsis*. RNA samples from floral buds at the endo- and eco-dormancy stages showed that the mRNA levels of *SAP30 FUNCTION-RELATED 1* (*AFR1*) and *SAP30 FUNCTION-RELATED 2* (*AFR2*) were significantly increased as the dormancy process concluded (Figure 4A). Another gene displaying a similar pattern, though not statistically significant, was the *DNA ONE ZINC FINGER PROTEIN* (*DOF*) mRNA. Conversely, the *ATP DEPENDENT HELICASE BRAHMA* (*BRM*) and *DNA One Zinc Finger Protein (DOF)* mRNA levels seem not to have changed as dormancy advanced. Expression levels of the homologous gene versions (TAIR’s ID in Supplementary Table S1) were determined in the leaves of 77-day-old miR396 transgenic Edi-0 individuals (Figure 4B). These determinations depicted increased levels for *BRM*, *DOF*, and *AFR2* genes. Conversely, *AFR1* levels decreased in these transgenic individuals while *BRM* and *DOF* showed an increase in their mRNA levels. *AFR2* gene expression increased in transgenic Edi-0 individuals.

The early flowering phenotype of transgenic Edi-0 lines overexpressing amiR396 prompted us to conduct further analyses of the key components of the flowering repressor complex *SVP* and *FLC*. As shown in Figure 4C, a reduction in *FLC* gene expression was observed in these transgenic lines, while *SVP* mRNA levels increased due to ectopic miR396 overexpression. Additionally, the expression of the integrator florigen *FT* was found to be elevated, although its homolog *TWIN SISTER OF FT* (*TSF*) and the flowering repressors *TERMINAL FLOWER 1* (*TFL1*) and *VERNALIZATION INSENSITIVE 3* (*VIN3*) did not show variations between wild-type and 77-day-old transgenic individuals.

## 3. Discussion

Recent studies have shown that miRNAs play a significant role in regulating different aspects of dormancy progression in sweet cherry floral buds [27,54,55]. These molecules were differentially expressed under field (regular seasonal) and controlled non-stop (continuous) chilling conditions, unveiling the involvement of developmental pathways, stress responses, and growth activities. These activities are expected to actively participate in the process leading to the onset of flowering. In the present work, we identified and mapped miRNA-target gene nodes that could represent active transcriptional and epigenetic regulations in this process, highlighting the miR396 molecule as an important player capable of inducing early flowering in a vernalization-requiring *Arabidopsis* ecotype.

Micro RNA396 belongs to a group of conserved miRNAs that regulate plant development and hormone signaling [37] by regulating transcription factors that control cell proliferation and organ growth in plants [35]. Different *GRF* genes have been described to be targeted by this miRNA depending on the plant species and development stage [38]. MiR396 also interacts with other miRNAs and phytohormones to modulate the *GRF*-mediated responses to environmental stress and developmental signals [39], suppressing the expression either of *GRF* genes or of *GRF*-interacting factors [35]. Therefore, miR396 can be considered to play an important role in the fine-tuning of the regulatory network associated with *GRF*s in plants. Our findings revealed additional involvements for this miRNA, suggesting its action on regulatory networks for genes associated with transcription and epigenetic processes. The expression of *AFR1* and *AFR2* genes during dormancy and/or ectopic expression of this molecule evidenced modulations of these genes, which are involved in fine-tuning regulatory processes associated with flowering. Some differences were observed in the expression pattern of these genes in floral buds at dormancy release and Edi-0 plants overexpressing amiR396. During the chilling period, the sweet cherry branches were kept at 4–6 °C in the dark for progressive chilling accumulation while Arabidopsis plants were cultivated under long photoperiods. The differences observed in miR396 target genes may be due to the different light conditions, as these genes possess light-responsive putative cis-acting regulatory elements in their 5′-upstream regions (Supplementary Table S3). The involvement of both *AFR*s in deacetylation events during flowering has been previously shown in *Arabidopsis* [56]. AFRs form deacetylation complexes with Histone Deacetylases (HDACs) (AFR1/AFR2-HDAC complexes) to modulate the acetylation level of FT chromatin at long days (LDs). In addition, a direct interaction of these AFRs with a MADS-domain transcription factor AGAMOUS LIKE 18 (AGL18) results in the recruiting of these proteins and presumably of AFR1/AFR2-HDAC complexes to *FT* chromatin specifically at the end of LDs, leading to histone deacetylation upon *FT* activation [56]. In floral buds we found both *AFR1* and *AFR2* mRNA levels significantly increased from endo- to eco-dormancy transition, suggesting an important involvement of this deacetylation machinery along the sweet cherry dormancy process.

In contrast, in *Arabidopsis* individuals overexpressing miR396 and exhibiting an early flowering phenotype, the induction of *AFR2* was confirmed, while *AFR1* transcript levels were decreased. This suggests that the modulation of histone acetylation and gene expression in response to environmental cues (LDs) by AFR-HDAC complexes on FT was either missed or overcome in these individuals. Supporting these previous observations are the results derived from the elevated FT transcript levels in the transgenic individuals, which were accompanied by unaltered *TSF* expression. *TWIN SISTER OF FT* acts redundantly with *FT* to promote flowering in the photoperiod pathway and is regulated by *CONSTANS* (*CO*).

Two other genes integrating the miR396 node and linked to modulatory activity involving chromatin remodeling and RNAi processes were investigated. A decreasing trend from endo- to eco-dormancy in the mRNA levels of the *BRM* ATPase gene, one of the two catalytic components of the switch/sucrose non-fermenting chromatin remodeling complex (SWI/SNF CRC), was observed. On the contrary, in the early-flowering *Arabidopsis,* we found increased *BRM* transcript levels associated with this phenotype. The complex SWI/SNF has been described to possess chromatin remodeling activity and, in addition, to participate in chromatin-independent pri-miRNA processing [57]. These results could also be due to a differential stage between both species, clearly stating that dormancy and flowering events involve these processes, although under different contexts, which probably in the transgenic model species resulted in accelerated or eliminated at the stage used for the molecular characterization (77-day-old individuals). In addition, this eventual unraveling of epigenetic events taking place in the early flowering *Arabidopsis* could also be illustrated by the determined *VIN3* mRNA levels, a chromatin remodeling protein that is induced by low temperatures to repress *FLC*, which resulted unaltered in these transgenic individuals.

An increasing trend for mRNA accumulation from endo- to eco-dormancy for a member of the *DOF* transcription factor family, a group of zinc finger proteins involved in plant development and stress responses, was consistently found in both floral buds and early flowering *Arabidopsis*. In *Prunus* spp., five *DOF* family members have been shown to have increased levels during dormancy in *P. persica* [58]. In this context, our findings agree with these antecedents, indicating active processes in the dormant buds. Consequently, increased *Arabidopsis DOF* mRNA levels could represent the active physiological condition found in these individuals, caused by permanent flowering, although showing an apparent reduced metabolic condition, as derived from the observation of plants with generally reduced structures such as leaves. In addition, involving both flowering and development, we found that *TFL1* mRNA levels were unaltered in the early flowering lines compared to wild-type *Arabidopsis* Edi-0 individuals, reinforcing the occurrence of a probable bypass for this *FT* inhibitor associated with inflorescence development.

### 3.1. Ectopic Expression of miR396 in Arabidopsis and Early Flowering

The ectopic expression of miR396 in the *Arabidopsis* Edi-0 ecotype led to premature flowering in the transgenic *Arabidopsis* individuals. The mechanisms modulating the expression of *SOC1* and *FT* respond to the MADS-box transcription factors *FLC* and *SVP* in the format of a repressor complex under non-inductive conditions [18,20]. When acting independently, these factors exhibit distinct spatial expression profiles in leaves and apices, determining specialized individual roles in flowering and plant development [20]. In this way, *SVP-FLC* repressor complex levels in miR396 overexpressing Edi-0 lines are expected to be dramatically decreased due to the lack of *FLC* transcripts, allowing for the observed early flowering phenotype.

*FLOWERING LOCUS C* transcript levels are reduced by exposure to winter cold (vernalization), allowing flowering to occur [59,60]. *FLOWERING LOCUS C* is a potent inhibitor of *FT*. During vernalization, the *FLC* promoter is remodeled and silenced by *VIN3*, allowing *FT* expression. Whereas Col-0 and Ler ecotypes have a non-functional FRI/FLC module, Arabidopsis ecotypes requiring vernalization express high FLC levels due to the functional FRI/FLC module, which are then repressed due to chilling accumulation [61,62]. Our results showed that the overexpression of miR396 in the vernalization-requiring Edi-0 was able to conduct early flowering that coincided with decreased *FLC* mRNA levels. The capability of miR396 suppressing the vernalization requirement for flowering has been recently indicated in *Agrostis stolonifera* associated with epigenetic regulation of *VRN* genes [36]. In *A. stolonifera*, *AstVRN1* (a MADS-box transcription factor gene that regulates vegetative to reproductive transition) and *AstVRN3* (ortholog of *FT*) were found to be induced by miR396 overexpression, whereas *AstVRN2* (a flowering repressor that encodes a CCT domain and zinc finger-containing protein not related to *FLC*) was repressed. As mentioned above, in Edi-0 individuals we did not observe increased *VIN3* transcript levels, suggesting a condition in which the overcoming of regular epigenetic events is caused by the ectopic expression of miR396.

*SHORT VEGETATIVE PHASE* transcription has been described as declining in the inflorescence meristem under inductive flowering conditions. However, *SVP* mRNA reappears shortly after in floral primordia, allowing this transcription factor to participate in additional processes involved in flower development [63,64]. Chorostecki et al. [65] defined a sequence conservation bioinformatic approach showing that the *SVP* gene could represent an eventual miR396 target by hosting a recognition site at the MADS box region. This activity was later demonstrated by Yang et al. [66] by analysis of the “leafy flower” symptoms caused by the peanut witches’ broom (PnWB) phytoplasma during infection of *Catharantus roseus*. In that work, indirect evidence suggested that, rather than miRNA-mediated cleavage, the *SVP* decrease was caused by miR396-mediated translation inhibition, which was consistent with the formation of abnormal flowers. Our results showed that the ectopic expression of miR396 led to increased *SVP* mRNA levels, a condition that can be attributed to the permanent flowering development and the involvement of *SVP* in these developmental processes. It is also worth noting that Hou et al. [67] also observed early flowering in the vernalization-independent *A. thaliana* Col-0 plants knocked out for miR396a and 396b genes. This early flowering phenotype was also correlated with the overexpression of *FT*, while the expression of other flowering-related genes remained unchanged. These results suggest that the regulatory network underlying the control of flowering relies on a complex array where the imbalance of mir396 expression can result in dramatic changes through a series of elements and mechanisms that require further elucidation.

### 3.2. Other microRNAs

Our results also showed an involvement of miR162. This miRNA is crucial in the regulatory network of plant adaptation to environmental stress that has been involved in stress-associated events such as stomatal conductance, drought response, and abscisic acid signaling in tomato [42] and *Arabidopsis* [68]. Micro RNA 162 targets the *DCL1* mRNA, leading in that way to the regulation of miRNA biogenesis and, eventually, to the generation of additional siRNA families. In the present work, we included Edi-0 transgenic lines overexpressing miR162 as a control situation of miR396. At present, these plants have shown an intermediate phenotype between wild-type and miR396 individuals (Supplementary Figure S3). The key role of this molecule in stress-like processes represents the regulation of processes involving genes such as *WRKY33*, *WRKY40*, *CNI1*, and *CML372* [42]. Interestingly, the regulatory node miR162-DCL1 can be regulated by several other stress-responsive miRNAs, such as ath-miR5021, miR413, miR5998, and miR162 itself [68]. As for miR396 materials, future research will be carried out with the already generated materials for miR162.

In addition, miR403 is a key regulator of the AGO2-dependent miRNA pathway in plants, suggesting a node (miR403-AGO2) as it has been described for miR168-AGO1 [69], which is in turn associated with the silencing and counter-silencing events taking place in some plant-virus interactions [70,71] and to plant development [72]. over the overexpression of miR403 in tomato showed flowering delay, leaf morphology, and resistance to ABA during germination phenotypes, causing miR156, miR159, and miR394 accumulation [73]. Among the possible targets, the *ABNORMAL INFLORESCENCE MERISTEM 1* (*AIM1*) gene is highlighted, which encodes the 3-hydroxyacyl-CoA dehydrogenase. The *AIM1* is an enzyme involved in β-oxidation and participates in both vegetative and reproductive development by regulating salicylic acid content and thus modulating the antioxidant system described as a hub of the dormancy process in *Prunus* spp. [11].

The finding of miR167 as a differentially expressed molecule in our study highlights the direct involvement of miR167 and auxin response factors (ARFs) during dormancy (node miR167-ARFs). MiR167 establishes an active crosstalk during different cell episodes, including flower development, which is achieved by targeting different Auxin Response Factors (*ARF*), including *ARF6* and *ARF8* [74,75]. In addition, interaction between miR167 and *ARF17*, a negative regulator of lateral development and nodulation, has also been established [74], stressing the relevance of this crosstalk. Moreover, miR167 interaction with other hormone pathways such as cytokinin, abscisic acid, ethylene, and jasmonic acid has also been found, revealing the impact of this involvement on various developmental processes and stress responses in plants [75].

Finally, miR166 targets *HD-ZIP III* transcription factors and has been described in shoot apical meristem formation, vascular differentiation, and leaf and root development. During stress, this miRNA has been associated with the management of abiotic stresses such as drought, salinity, and temperature fluctuations [76].

### 3.3. Final Considerations

An increase in the expression of miR396 in sweet cherry floral buds after fulfillment of chilling requirements. Additionally, our results have shown that an artificial miR396 molecule is able to induce an early-flowering condition in Arabidopsis Edi-0 individuals without vernalization. This result strongly indicates that this molecule may induce flowering in annual and perennial plant species. As is known, small RNAs can travel systemically through the plant. The use of transgenic miR396 overexpression rootstocks could be a potential application in fruit tree breeding aiming to obtain an early flowering and/or low chilling requirement grafted varieties. Ectopic application of artificial miR396 through spraying could also be a biotechnological approach that allows for effective field management considering the current challenges imposed by the climate change scenario.

However, flowering induction is a result of different levels of regulation across different cell types. Moreover, environmental cues such as photoperiod, temperature, and abiotic stress conditions may have an impact not only on flowering but also on other developmental and phenological stages. Indeed, we observed that overexpression of miR396 also affects vegetative development in Arabidopsis. These plants did not display the typical rosette structure of Brasicaseae. Further investigation is needed, including additional techniques that would allow detailed analysis of in situ epigenetic reprogramming and hormonal signaling, for example, to fully understand flowering transition in woody fruit species.

## 4. Materials and Methods

### 4.1. ‘Regina’ Material, Sampling, and Chilling Requirement Determination Under Forcing Conditions

Adult (8–10-year-old) sweet cherry ‘Regina’ trees, which were part of the INIA Sweet Cherry Breeding Program collection located at the Rayentué Experimental Station, O’Higgins Region of Chile (34°19′17″ S 70°50′4.2″ W), were used. Samples of corresponding branches bearing floral buds were obtained from this orchard at the beginning of autumn 2021 (April in the Southern hemisphere) and subjected to continuous chilling as described by Soto et al. [27]. Briefly, the collected branches were transported to the laboratory, disinfected, separated into lots of 4–5 branches, and stored at 4–6 °C for progressive chilling accumulation. Branches with differential cold accumulation (200, 1160, and 1700 chilling hours (CH)) were incubated in water pots and placed under forcing conditions at 25 °C under a 16/8 h day/night photoperiod. After 14 d, the phenological status of floral buds was scored, and chilling requirement was considered fulfilled when at least 50% of the buds burst (BBCH 51 stage; [77]). In parallel, branches from the same CH time point were used as a source of floral buds, from which these structures were cut off from branches immediately before the stick heat activation process and immediately frozen in liquid nitrogen and stored at −80 °C for small RNA isolation and sequencing.

### 4.2. ‘Regina’ Total and Small RNA Isolation

Floral bud samples from 8 to 10 different branches were subjected to RNA isolation following the procedures described by Sánchez et al. [78]. Processed samples (100 mg) allowed for low- and high-molecular-weight RNA isolates from samples (LMW and HMW, respectively), which were stored at -80 °C for further studies.

### 4.3. Yield and Quality Analysis of Isolated RNA Fractions

Between 0.5 and 1 µL of total RNA was used to test the quality and quantity using a QuantiFluor™-ST Fluorometer (Promega, Madison, WI, USA), and the size/distribution/integrity of fragments was evaluated using an Agilent 2100 Bioanalyzer system (Agilent, Santa Clara, CA, USA) using standards and according to manufacturer instructions for these samples. For accurate small RNA quantification, a fluorometric assay was assessed by using the Quant-iT™ RiboGreen^®^ RNA Assay Kit (Thermo Fisher Scientific, Waltham, MA, USA) according to the manufacturer’s protocol.

### 4.4. Library Construction and Sequencing

Small RNA libraries were built and sequenced on an Illumina HiSeq using paired-end sequencing of 150 bp in length at the Genoma Mayor Sequencing Facility Center (Universidad Mayor, Huechuraba, Santiago, Chile). Since paired-end reads do not contribute useful information in small RNA-seq studies, only the forward reads were used for further analyses. The quality of the raw small RNA-seq data was assessed using FastQC. Adapters were removed, and sequences were trimmed and filtered using Cutadapt v4.9 [79].

### 4.5. Differentially Expressed (DE) Micro RNAs and Target Prediction

A dataset generated from sRNASeq experiments was generated for miRNAs. Reads were mapped to the *Prunus avium* cv. Tieton reference genome v2.0 [80] using Shortstack [81]. To define DE miRNAs, the sRNAs identified by Shortstack were annotated using the information available in miRBase and Plant small RNA genes [82] databases. The table of counts was obtained for all the clusters identified by Shortstack. The R package DEseq2 [83] was used to identify the differentially expressed small RNAs among the conditions. Counts were normalized using the median of ratios, and low-count tags were discarded according to the package pipeline. MicroRNAs were tested for differential expression compared to the first stage of chill accumulation (CR 200) using a likelihood ratio test (LRT). *p*-values were adjusted using the Benjamini–Hochberg method, and a *p*-adj value < 0.05 was used as a filter. The targets of the identified miRNAs were predicted using the psRNAtarget web interface [84]. A cut-off value of E = 3.0 was selected for miRNA target sequence matching to find potential degradation by cleavage.

### 4.6. Experimental Determination of Putative Target Genes

For target gene amplification, the HMW RNA fraction was treated and checked for DNA contamination as before, and first-strand cDNA synthesis was performed using oligo-dTs in a reverse transcription step. Expression patterns were assessed using qRT-PCR. Briefly, each reaction was run in triplicate with 1 µL of cDNA in a 20 µL final volume using 0.6–0.8 µM of the corresponding primers (Supplementary Table S4) and 1X Eva Green master mix (Biotium, Fremont, CA, USA). The analysis was performed using a G8830A AriaMx Real-time PCR System (Agilent Technologies, Santa Clara, CA, USA). The 2-ΔΔCT method was used for relative quantification of miRNA and normalized using sRNA obtained from the sRNA sequencing of cherry flower buds, noted as precursor_393 (see Supplementary Table S4), and using the EF1α transcript for expression of target genes [85].

### 4.7. Artificial microRNA (amiRNA) Synthesis

Artificial versions of miR396 and miR162 (amiR396 and amiR162, respectively) were generated using the *Vitis vinifera* miR319e backbone and the long primer strategy described by Castro et al. [86]. Briefly, a two-step PCR was used to generate each pre-amiR319e-derivative; in the first reaction, a mixture contained 0.5 U of KAPAHiFi (KAPA Biosystems, Wilmington, MA, USA), 0.3 mM dNTPs (Promega Corporation, Madison, WI, USA), 1X Fidelity Buffer with MgCl_2_ and 50 ƿmol of each long-primer for each amiRNA (Supplementary Table S4). The reaction had a final volume of 25 µL. The thermal profile was as follows: 94 °C for 2 min; 10 cycles of 94 °C for 15 s, 55 °C for 30 s, and 72 °C for 15 s; and a final elongation at 72 °C for 30 s. The amplification product (i.e., pre-amiR396 or pre-amiR162) was subjected to a second round of PCR to facilitate attB signal completion for subcloning into the pDONR207 vector. This step was carried out via a reaction that contained 0.5 U of KAPAHiFi (KAPA Biosystems), 0.3 mM dNTPs (Promega Corporation), 1X Fidelity Buffer with MgCl_2_, 7.5 ƿmol of each primer (attB-F and attB-R; Supplementary Table S4), and 10 µL of the corresponding pre-amiRNA. The reaction had a final volume of 25 µL. The thermal profile was as follows: 94 °C for 1 min; 5 cycles of 94 °C for 15 s, 45 °C for 30 s, and 72 °C for 20 s; 20 cycles of 94 °C for 15 s, 60 °C for 30 s, and 72 °C for 20 s; and a final extension at 72 °C for 1 min. The final amplification product was resolved with a 1.5% UltraPure Agarose (Thermo Fisher Scientific) gel and visualized via ethidium bromide staining. The band of interest was recovered by gel extraction using the Zymoclean Gel DNA Recovery Kit (Zymo Research) according to the manufacturer’s instructions. The purified amplicons were cloned into the donor vector through recombination using the Gateway BP Clonase System (Thermo Fisher Scientific). An aliquot of each pre-amiRNA (150 ng) was recombined with 50 ng of the pDONR207 vector to generate the BP recombination reaction, according to the manufacturer’s protocol. From this BP reaction, an aliquot (1 μL) containing the resulting vector (pDONR-pre-amiR392 or pDONR-pre-amiR162) was used to transform *Escherichia coli* One Shot TOP 10 (Thermo Fisher Scientific) competent cells according to the manufacturer’s instructions. The transformed cells were selected via incubation in LB medium supplemented with 15 mg/L of gentamycin overnight at 37 °C. The selected clones were grown in 5 mL of LB medium supplemented with 100 mg/L of spectinomycin at 37 °C overnight with shaking at 180 rpm; the cultures were centrifuged at 8000× *g* and subjected to plasmid DNA extraction using the Zyppy Plasmid Miniprep Kit (Zymo Research). The plasmid DNA was checked by PCR and restriction enzyme analysis. For PCR, the primers amiRNA-F and amiRNA-R (Supplementary Table S4) were used with the following thermal profile: 94 °C 2 min; 35 cycles of 94 °C for 15 s, 60 °C for 30 s, and 72 °C for 20 s; and a final extension at 72 °C for 1 min. Restriction analysis was carried out by incubating 10 U of Sac I (New England Biolabs, Ipswich, MI, USA), 1X NEB1.1 (New England BioLabs), 1 µg/µL of purified BSA, and 500 ng of plasmid for a final volume of 30 µL. Restriction was carried out via incubation overnight at 37 °C. The restriction assay was resolved on a 1.5% agarose gel and visualized via ethidium bromide staining. The selected pDONR-pre-amiRNA plasmids were confirmed by sequencing (Macrogen, Seoul, Republic of Korea).

### 4.8. Expression Vector for Pre-amiRNAs and Agrobacterium Clones

Each vector pDONR-pre-amiRNA was recombined into the Gateway expression vector pGWB502 [87]. Recombinations were carried out by mixing 150 ng of pDONR-pre-amiRNA and 150 ng of pGWB502, using the Gateway LR Clonase System (Thermo Fisher Scientific) according to the manufacturer’s instructions. The recombination mix (1 μL) was used to transform *E. coli* One Shot TOP 10 competent cells, and the positive clones were selected on LB medium supplemented with 100 mg/L of spectinomycin. The positive clones were verified via PCR using the pre-amiRNA-F and pre-amiRNA-R primers (Supplementary Table S4) and restriction analyses using 10 U of the *Sac I* enzyme (New England Biolabs, USA) and 1X NEB2, as indicated for *Sac I*. The resulting vectors were denominated pGWB-pre-amiR396 and pGWB-pre-amiR162 and confirmed by sequencing (Macrogen) and used in the transformation of *Rhizobium radiobacter* (*Agrobacterium tumefaciens*) GV3101 strain by electroporation using Gene Pulser equipment (Bio-Rad, Hercules, CA, USA) using the following conditions: 1.25 V, 400 ohms, 25 μF.

### 4.9. Genetic Transformation of A. thaliana Ecotype Edi-0

Arabidopsis Edi-0 seeds (Arabidopsis Biological Resource Center stock number #CS1122) were used. Seeds were put directly onto wet soil and grown for 6 weeks at 21 °C, 16 h light/8 h dark. Once the rosette was developed, the plants were transferred to a 4 °C refrigerator with dim light and kept there for 4 weeks to accelerate the vernalization process and then returned to standard conditions (21 °C, 16 h light/8 h dark) for 4–6 weeks until bolting. Gene transfer was performed by floral dip using *Rhizobium radiobacter* (formerly *Agrobacterium tumefaciens*) strain GV3101 clones harboring the amiRNA expression vectors, according to the protocol previously described [88]. Obtained seeds were placed in Petri dishes containing 25 mL of semisolid MS [89] supplemented with 2 g/L of Phytagel (Sigma, Livonia, MO, USA), 25 g/L sucrose, 100 mg/L cefotaxime, and 100 mg/L hygromycin for 14 d for germination and subsequent antibiotic selection.

### 4.10. Genetically Modified Arabidopsis Populations

Twenty self-pollinated individuals for each generation were selected randomly to obtain a T3 generation for each construct. All individuals were grown at 21 °C, 16 h light/8 h dark, and evaluated in the T1 and T3 subpopulations. Genomic DNA (gDNA) was purified from the leaves of 11-week-old plants using the extraction protocol described by Edwards et al. [90] and quantified using the Bio Spec-nano^®^ computer (Shimadzu, Carlsbad, CA, USA). Transgene insertion was evaluated by PCR amplification of a 104-base pair (bp) fragment of the endogenous gene EF1α (EF-1ALPHA, TAIR_ID: AT1G18070) using AtEf1α-S1 and AtEf1α-A1 primers. The presence of the amiRNA expression cassette was evaluated by amplifying a 687 bp fragment, and the persistence of *R. radiobacter* was controlled by amplifying a 391 bp fragment corresponding to the *virG* gene (GenBank: NG_034482.1) using virG_Fw and virG_Rv primers (Supplementary Table S4). PCRs were performed according to conditions previously described [91].

### 4.11. Transformed Arabidopsis Total and Small RNA Isolation

Plant samples from transgenic and wild-type individuals were subjected to RNA isolation following the same procedures described above. Plant samples (100 mg of leaf tissue) from transgenic and wild-type individuals were subjected to RNA isolation using Trizol (ThermoFisher Scientific) following the manufacturer’s protocol. The total RNA was used to isolate small RNA fractions according to procedures described by Sánchez et al. [78] and the same procedure described above for RNA extracition of ‘Regina’ floral buds samples. Processed samples (100 mg) allowed LMW and HMW RNA isolates, which were stored at −80 °C for further studies.

### 4.12. Experimental Determination of microRNAs and Target Genes in Arabidopsis

For small RNA detection, 1 µg of LMW RNA was incubated with *DNAse I* (Thermo Fisher Scientific) and checked for genomic DNA contamination by PCR amplification of an amplicon spanning through exons 4 and 5 of the *GLYCERALDEHYDE-3-PHOSPHATE DEHYDROGENASE C2* (*AtGAPC*) gene [92]. Complementary DNAs for miRNAs were obtained from these DNA-free RNA extracts using stem-loop-based qRT-PCR [93]. The structural sequence of the primer required for the stem loop had the sequence 5′-GTCGTATCCAGTGCAGGGTCCGAGGTATTCGCACTGGATACGACNNNNNN-3′, in which the positions marked by N were miRNA specific (6-base overhang for specific interactions between the primer and the target miRNA) (Supplementary Table S4). Synthesis was carried out using the Superscript First-Strand kit (Thermo Fisher Scientific), according to the manufacturer’s protocol, in which 2 pmole of each primer for stem-loop qRT-PCR (Supplementary Table S4), 1 μL of 10 mM dNTP mix, and nuclease-free water to a final volume of 12 μL were used. For target gene amplification, the HMW RNA fraction was treated and checked for DNA contamination as before, and first-strand cDNA synthesis was performed using oligo-dTs in a reverse transcription step. Expression patterns were assessed using qRT-PCR. Briefly, each reaction was run in triplicate with 1 µL of cDNA in a 20 µL final volume using 0.6–0.8 µM of the corresponding primers (Supplementary Table S4) and 1X Eva Green master mix (Biotium, Fremont, CA, USA). The analysis was performed using a G8830A AriaMx Real-time PCR System (Agilent Technologies, Santa Clara, CA, USA). The 2-ΔΔCT method was used for relative quantification of the transcript abundance of the target genes, which was normalized through the measurement of the Ef1α transcript.

## Figures and Tables

**Table 1:** Statistics of the quality assessment of the libraries, alignment, and reads mapped to the ‘Tieton’ sweet cherry reference genome.

Condition	Raw Reads	HQ Reads	Mapping to Reference Genome (%)	21-nt	22-nt	23-nt	24-nt
Para- dormancy (200 CH)	24,078,331	18,843,039	87.8	2,215,658	1,932,853	1,727,362	3,717,064
Endo- dormancy (1160 CH)	25,413,001	17,044,809	88.7	2,035,231	1,723,603	1,470,882	2,667,987
Eco- dormancy (1700 CH)	23,798,208	15,740,688	88.1	2,242,584	1,676,026	1,260,979	2,605,945

**Table 2:** Role of miRNA families and target functions described in other species.

miRNA	Target	Target Function	Species	References
miR162	DCL1	Regulate miRNA biogenesis. Involved in the low night temperature responsive pathway by indirectly regulating stomatal conductance and photosynthesis	*Arabidopsis* and *Solanum*	[41,42]
miR167	ARF	Development of male organ, roots, stems, leaves and flowers, flowering time, embryonic development, seed development and stress	*Arabidopsis* and *Oryza*	[43,44,45,46]
miR396	GRF	Cell proliferation in leaves, disease resistance, somatic embryogenesis, grain size, and panicle branching	*Arabidopsis*, *Medicago*, and *Oryza*	[35,47,48,49,50,51,52]
miR403	AGO2	miRNA metabolism	*Arabidopsis*	[53]

**Table 3:** Phenotypic characterization of Edi-0 Arabidopsis transgenic lines overexpressing an artificial miR396 molecule.

Genotype/Plant *	Flowering Day (After Sowing)	Rosette Leaves at Flowering	Stemloop PCR/Sequencing **
WT (Edi-0)/p1	207	136	−/n.a.
WT (Edi-0)/p2	209	140	−/n.a.
WT (Edi-0)/p3	209	124	−/n.a.
WT (Edi-0)/p4	210	110	−/n.a.
WT (Edi-0)/p5	210	133	−/n.a.
396 T2 ^a^/p1	49	3	+/amiR396
396 T2/p2	61	4	+/amiR396
396 T2/p3	88	2	+/amiR396
396 T2/p4	60	0	+/amiR396
396 T2/p5	61	9	+/amiR396
162 T1/p1	146	40	+/amiR162

* Individual selected from the indicated genotype. ^a^ Transgenic generation. ** Artificial miRNA detection by stem-loop PCR/sequencing of the amplified stem-loop-generated amplicon. + positive; − negative; n.a. not applicable.

## Data Availability

Data are contained within the article and Supplementary Materials listed above.

## Supplementary Materials

The following supporting information can be downloaded at: https://www.mdpi.com/article/10.3390/plants14060899/s1: Figure S1: Development of Arabidopsis Edi-0 wild-type plants; Figure S2: Development of Arabidopsis Edi-0 transformed with the artificial miR396 molecule; Figure S3: Different T1 individuals generated from miR396 Edi- transformation assays. T1 individuals arising from new transformation rounds exhibit various early-flowering phenotypes. Some of these individuals have replicated the absence of rosette structure (*). Bars indicate approximately 1 cm.; Figure S4: Development of Arabidopsis Edi-0 transformed with the amiR162 molecule; Figure S5: Different T1 individuals were generated from miR162 Edi-0 transformation assays. T1 individuals from new transformation assays have been obtained. Materials are shown approximately 70 days post-germination. Bars indicate approximately 1 cm. Table S1: Differentially expressed miRNAs and their respective putative target genes; Table S2: qPCR data and statistical processing; Table S3: Putative cis-acting regulatory elements in the 5′-upstream region of *BRM*, *DOF*, *AFR1*, and *AFR2 Prunus avium* genes; Table S4: Primers used in this work.
