# Supplementary figures and images for: Artificial Sweet Cherry miRNA 396 Promotes Early Flowering in Vernalization-Dependent Arabidopsis Edi-0 Ecotype
Source: Plants (Basel). 2025 Mar 13;14(6):899. doi: 10.3390/plants14060899 (PMC11945767; doi:10.3390/plants14060899)

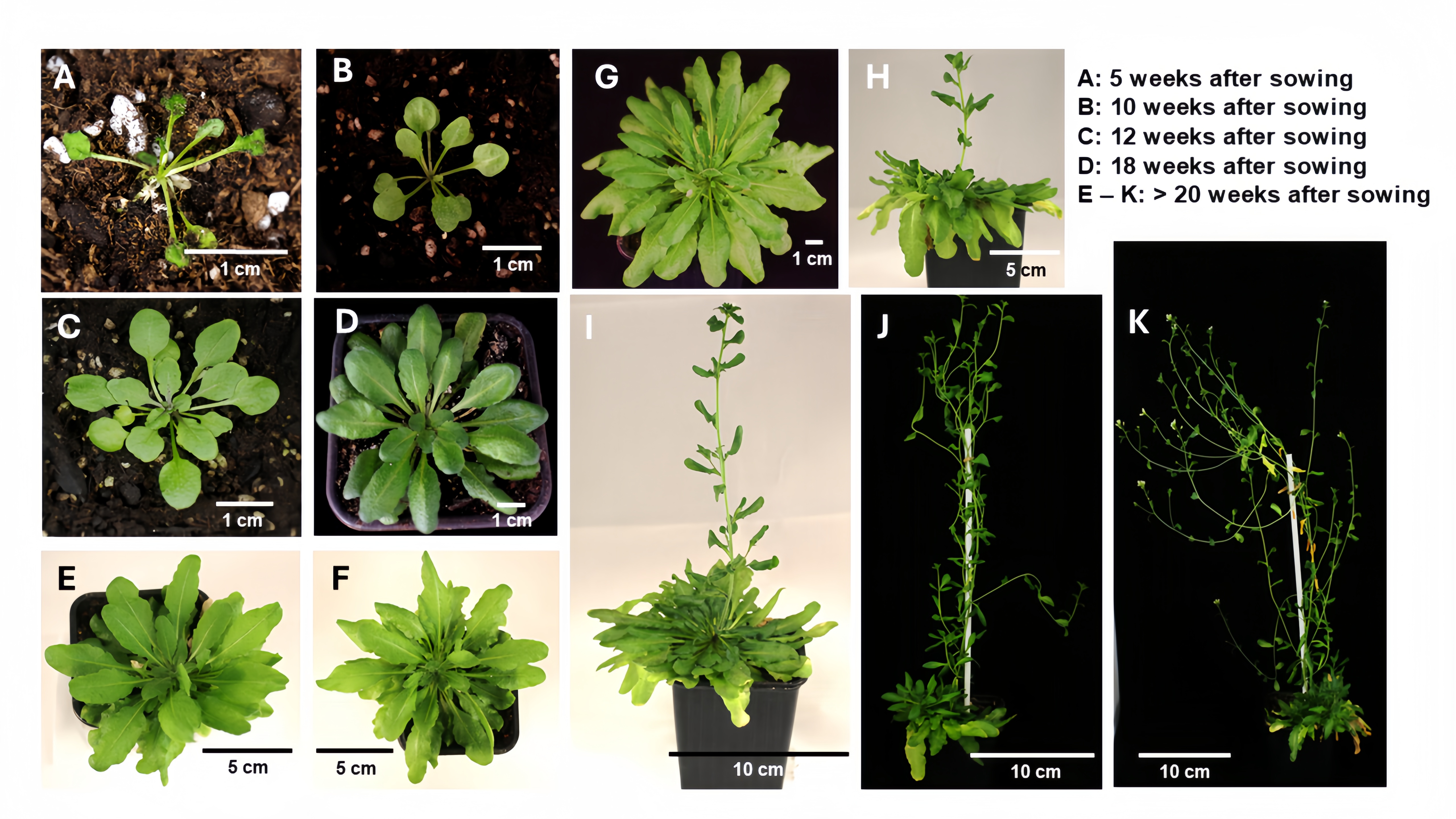

Supplement: Supplementary file 1 [file plants-14-00899-s001.zip › Supplementary Figure S1.jpg]

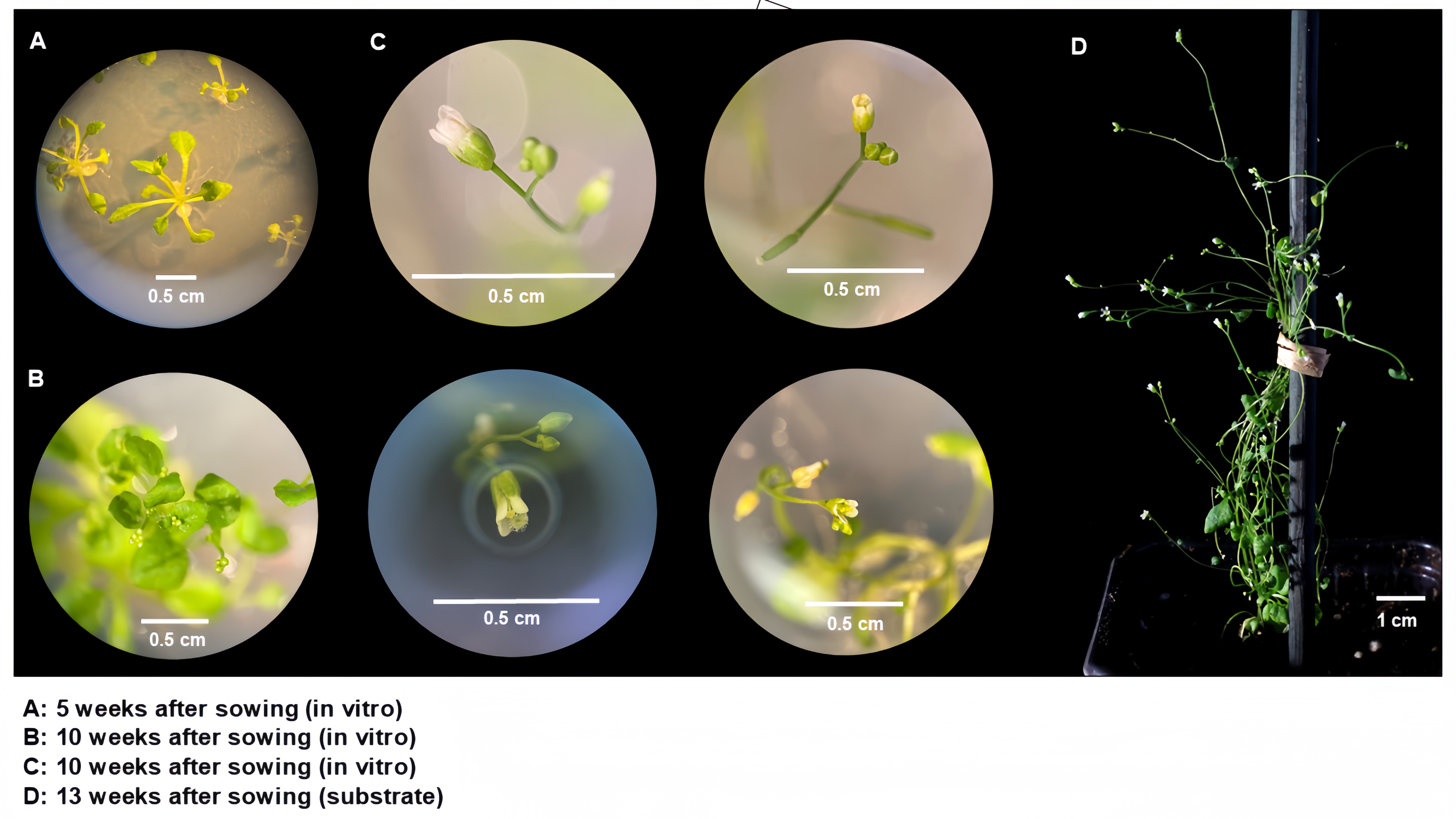

Supplement: Supplementary file 1 [file plants-14-00899-s001.zip › Supplementary Figure S2.jpg]

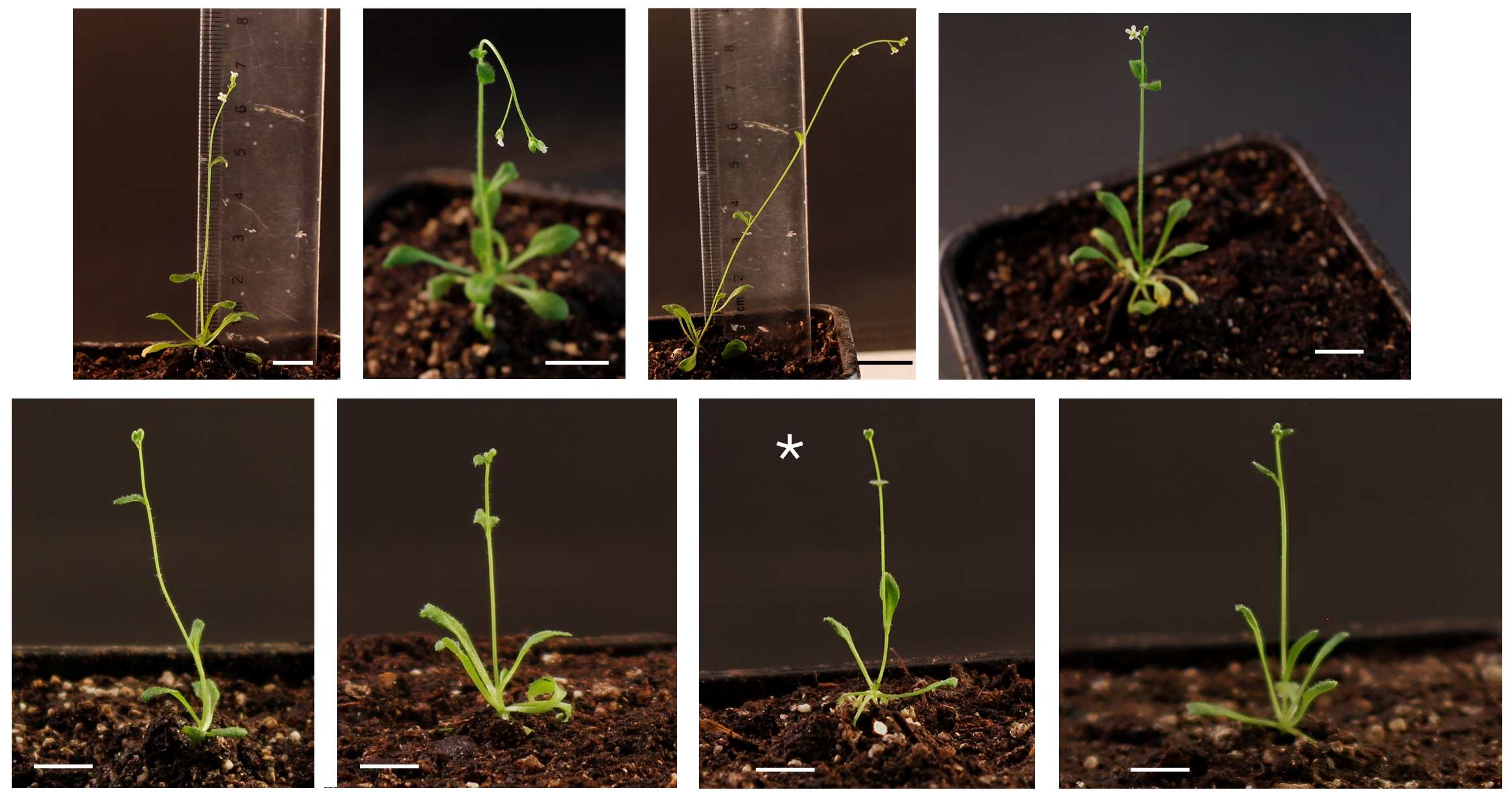

Supplement: Supplementary file 1 [file plants-14-00899-s001.zip › Supplementary Figure S3.png]

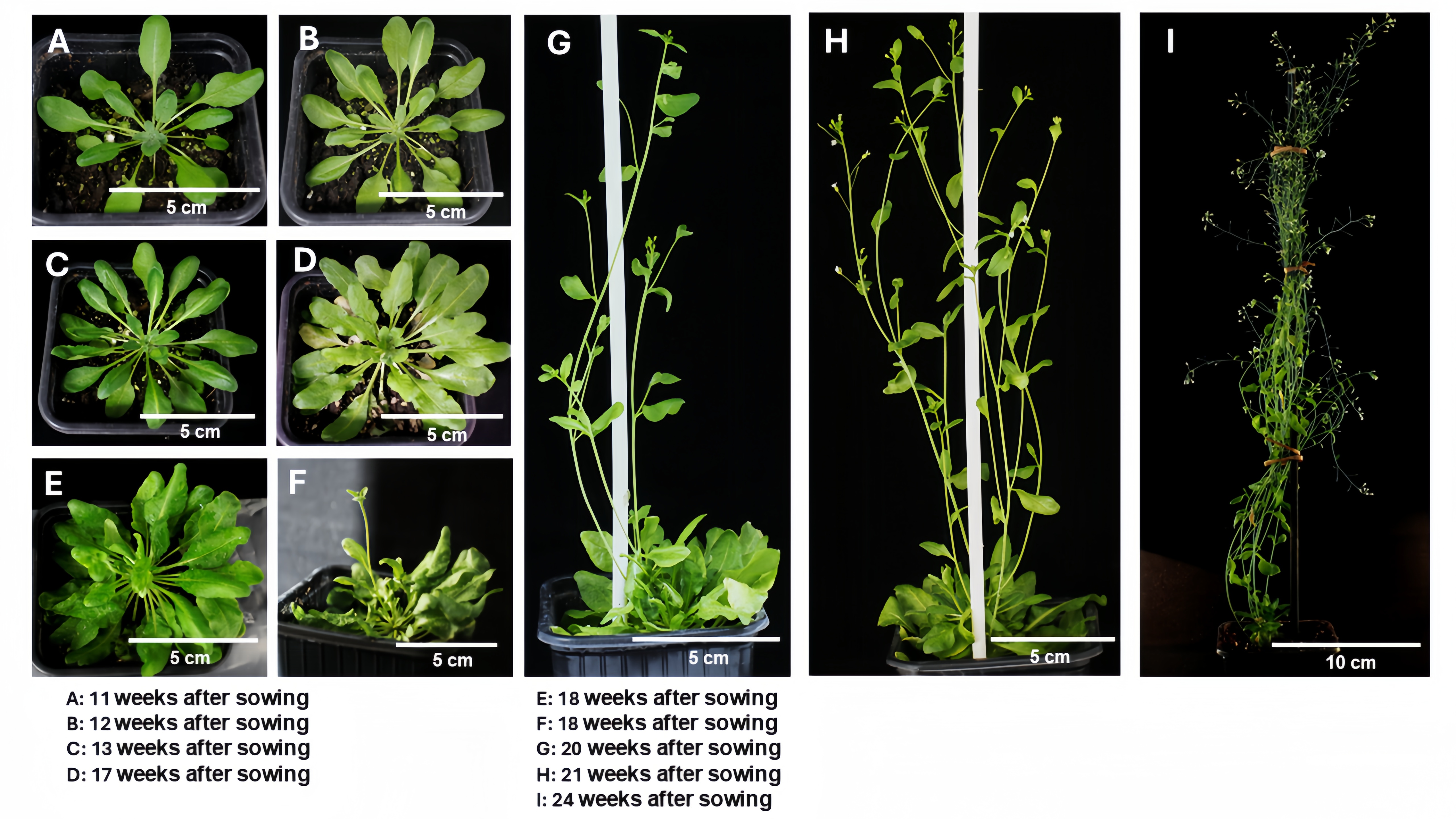

Supplement: Supplementary file 1 [file plants-14-00899-s001.zip › Supplementary Figure S4.jpg]

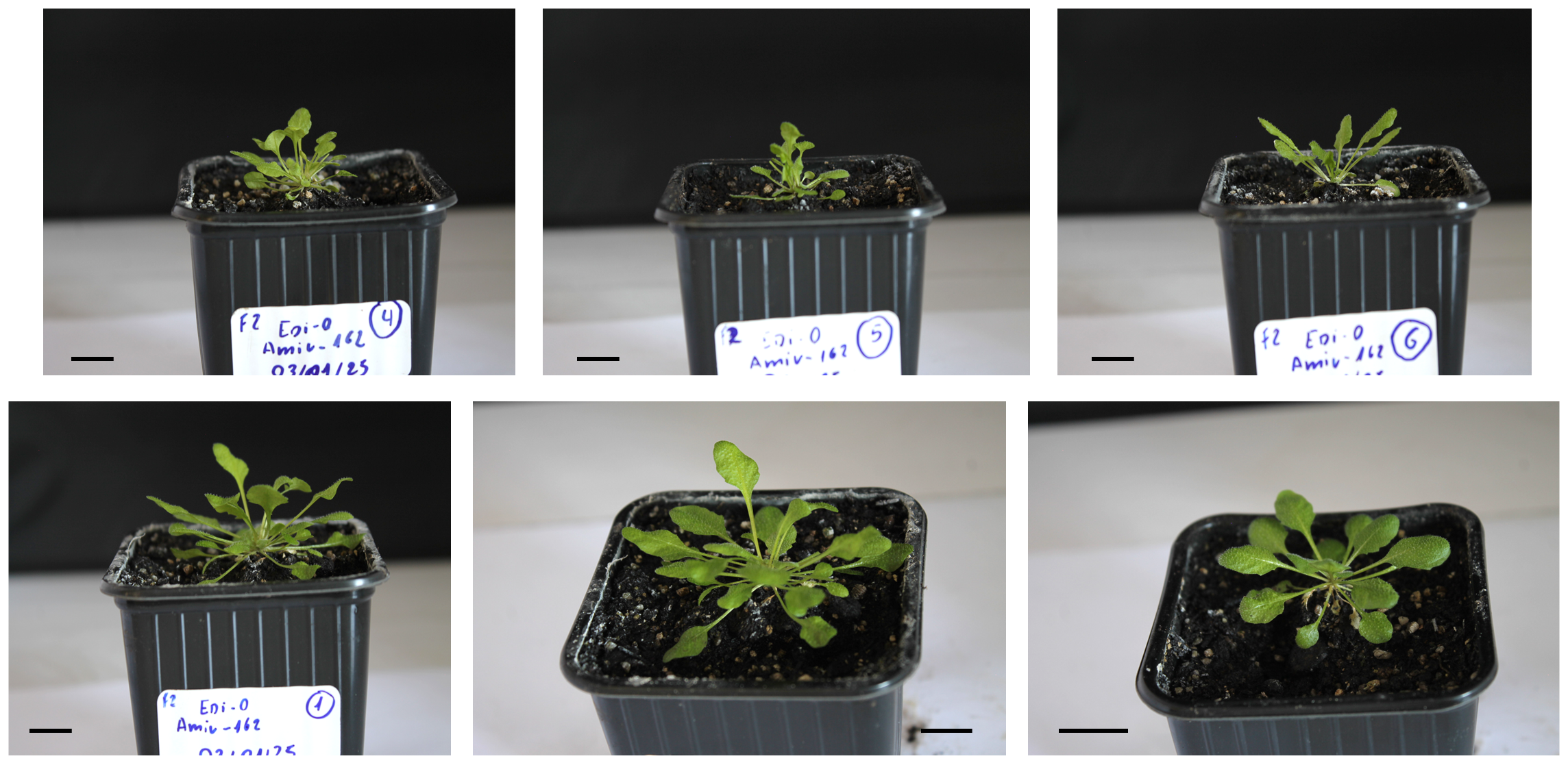

Supplement: Supplementary file 1 [file plants-14-00899-s001.zip › Supplementary Figure S5.png]
